# A Single-Centre Analysis of Surgical Techniques for Myelomeningocele Closure: Methods, Outcomes, and Complications

**DOI:** 10.3390/clinpract14050162

**Published:** 2024-09-29

**Authors:** Alina Roxana Cehan, Dorin Constantin Dorobanțu, Corina Ionela Tamas, Vlad Dimitrie Cehan, Flaviu Tamas, Adrian Balasa

**Affiliations:** 1 Plastic and Reconstructive Surgery, Emergency Clinical County Hospital of Targu Mures, 540136 Targu Mures, Romania; alina.hurghis@yahoo.com (A.R.C.);; 2 Plastic and Reconstructive Surgery, George Emil Palade University of Medicine, Pharmacy, Science, and Technology of Targu Mures, 540136 Targu Mures, Romania; 3 Neurosurgery, George Emil Palade University of Medicine, Pharmacy, Science, and Technology of Targu Mures, 540139 Targu Mures, Romania; 4 Neurosurgery, Emergency Clinical County Hospital of Targu Mures, 540136 Targu Mures, Romania; 5 Anesthesiology and Critical Care Clinic, Emergency Clinical County Hospital of Targu Mures, 540139 Targu Mures, Romania

**Keywords:** myelomeningocele, dysraphism, Limberg flap, VY flap, direct suture

## Abstract

(1) Background: Neural tube defects are a prevalent cause of congenital malformations, myelomeningocele (MMC) being the most severe form. This study evaluates the clinical outcome and postoperative-associated complications following MMC surgical closures, focusing on the following three techniques: direct suture (DS); VY advancement flap (VYF); and Limberg flap (LF). (2) Methods: A retrospective observational study was conducted from March 2015 to February 2023, and the inclusion criteria were newborns who underwent lumbosacral MMC within 24 h of birth. (3) Results: Out of 20 cases, 45% underwent DS closure; 25% underwent VY-F closure; 15% underwent LF closure, and 15% (*n* = 3) underwent combined flap closure. A significant statistical correlation was observed between intracranial hypertension (IH), the need for external ventricular drainage (EVD), and DS closure type. In the DS group, 60% of patients required EVD (*p* = 0.041), and 90% had IH (*p* = 0.027). CSF fistula was present in 40% of LF cases and 30% of DS cases, while wound dehiscence was observed in 60% of LF cases and 30% of DS cases. (4) Conclusions: Our study demonstrated that DS was linked to higher rates of complications. The VY-F is the safest method for closing MMC defects.

## 1. Introduction

Neural tube defects, the second most frequent cause of congenital malformations, constitute a spectrum of congenital anomalies, including cranial defects and open or closed spinal dysraphism. Myelomeningocele (MMC) is the most common and severe form of open spinal dysraphism (spina bifida aperta), with a global incidence of one to two cases per 1000 live births. MMC generally forms during embryonic development due to the incomplete closure of the spinal neural tube in the first month of pregnancy. This leads to the exposure of neural tissue or meninges, forming a placode that protrudes at the affected vertebral level. The vertebrae lack a neural arch at this level, thus being incomplete. MMC is often associated with various neurological deficits below the level of the involved vertebral segment. This condition can lead to severe morbidity and multiple disabilities, with MMC being among the top 10 causes of neonatal mortality. The prognosis is often unfavourable if the diagnosis is established late or therapeutic intervention is not undertaken [1].

Currently, the standard management of MMC includes postnatal surgery. Currently, Cochrane does not recommend prenatal surgery, with the exception of double randomised controlled studies [2]. Fetal surgery has become a common treatment method for managing MMC. Postnatal surgery is performed within the first 48 h after delivery, following a careful analysis of basic imaging studies and a thorough clinical examination, aiming to close the spinal lesion, therefore minimising the risk of infection. This pathology was previously associated with the need for a ventriculoperitoneal (VP) shunt in almost all cases of thoracic MMC, in up to 85% of cases where the lesion is at the lumbar level, and for approximately 70% of cases of sacral MMC. Studies conducted over the last two decades reveal a much better percentage regarding the need for shunt placement. For example, a study conducted in Lyon shows that only 25% required a ventriculoperitoneal shunt, while a study conducted in Hamburg-Altona presents a slightly higher percentage of 41%. Considering these values associated with postnatal surgery and consulting the MOMS II review, which presents a shunt percentage of 49% in prenatal surgery, we can assert that, at present, shunting in prenatal surgery is not better than in postnatal surgery. In recent years, ventriculocisternostomy with choroid plexus coagulation has become an alternative in treating hydrocephalus associated with MMC for selected cases. The results obtained on choroid plexus coagulation are mixed and still under debate; further studies are needed [3,4,5,6].

Fetal surgery is performed between 19 and 25 weeks of gestation, aiming at an early repair of the spinal lesion to prevent ongoing deterioration and improve clinical outcomes. Potential benefits include reducing the need for a VP shunt (this benefit is insignificant because recent studies show a similar percentage regarding the need for a VP shunt) and improving motor function. On the other hand, it is associated with a significant risk of premature birth and other surgical complications for both the mother and the fetus. The randomised controlled Management of Myelomeningocele Study (MOMS) shows that the tethered cord rate, inclusion of cysts, and dermoid formation are up to tenfold higher in prenatal surgery than postnatal [7,8,9].

There are still limited data regarding spinal dysraphism’s epidemiology, surgical treatment, management of complications, integration of these patients into society, and their quality of life. Regarding the closure of an MMC defect, it is considered that if the defect is minor, direct suture (DS) can be performed, and if the defect is large, various other types of reconstruction are performed. According to the literature, in most cases, DS is performed. In 25–35% of cases, various reconstruction techniques are used to close the skin and soft tissue defects, such as local or regional flaps of multiple compositions (muscular, fascial, cutaneous, or combined) or their variants and even skin grafts [10,11,12].

In the present study, we aim to report our eight-year experience with surgical techniques for closing MMC defects, analysing the three most common closure techniques used in our service (DS, VY advancement flap [VY-F], and Limberg flap [LF]), the results obtained, and the associated complications.

## 2. Materials and Methods

This is a retrospective, observational, single-centre study that included newborns (NBs) diagnosed with open spinal dysraphisms (MMC) who underwent surgery within the first 24 h after birth in the Neurosurgery Department, in collaboration with the Plastic Surgery and Reconstructive Microsurgery Unit, at the Clinical Emergency County Hospital of Targu-Mures, Targu-Mures, Romania. The observational period during which this study was conducted is 8 years, starting from March 2015 up to February 2023. All MMC surgical interventions were carried out collaboratively by our senior authors (A.B. and D.D.). This study was conducted in accordance with the Declaration of Helsinki, and the protocol was approved by our university’s Ethics Committee.

### 2.1. Inclusion and Exclusion Criteria

The inclusion criteria include the following: the presence of fistulated/non-fistulated MMC localised in the lumbosacral region, NBs operated within the first 24 h, the presence of basic imaging investigations (preoperative cranial and spinal computer tomography [CT] scans and postoperative cranial and spinal magnetic resonance imaging [MRI] and transfontanelar echography), and patients who underwent one of the following defect closure methods: DS; VY-F; or LF. The exclusion criteria include MMC localised at the thoracic level, surgery performed after 24 h for various reasons, and incomplete documentation regarding patient clinic or essential imaging.

### 2.2. Description of the Typical Surgical Technique

**Figure 1:**
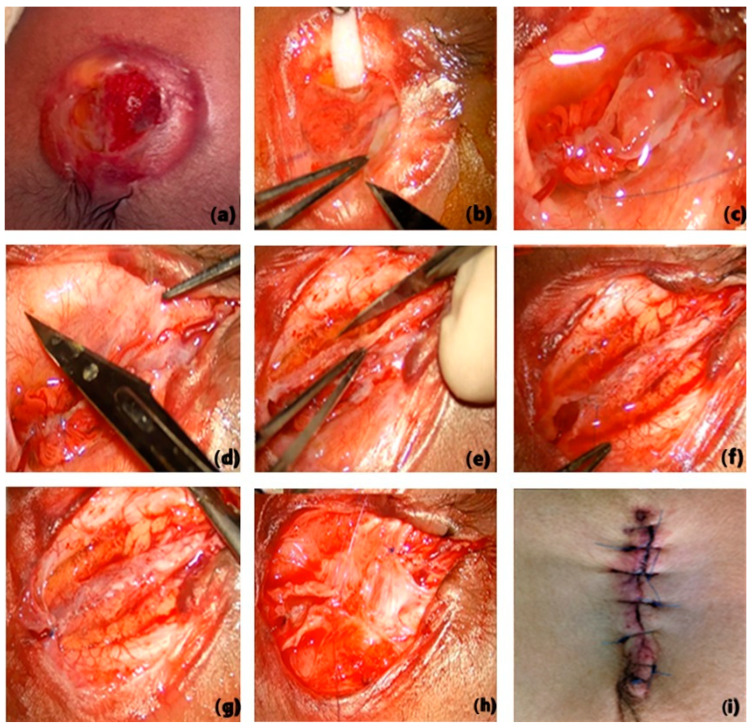
Surgical steps for MMC closure (**a**–**i**).

Under general anaesthesia, NBs were positioned in the prone position with the head slightly lower than the back to reduce cerebrospinal fluid (CSF) loss. The MMC was rinsed with warm sterile saline, and the surrounding skin was cleaned with Betadine. The surgical procedure was performed under an operating microscope (Leica OHX, Leica Microsystems, 35578 Wetzlar, Germany). A generous area of skin was exposed to allow for extensive mobilisation of the flaps. The neural placode was exposed, and an incision was made at the placode. In the wall of the meningeal sac, roots returning to the spinal canal were mobilised, and some nerve elements terminating in the meningeal sac were sacrificed. Subsequently, the edges of the neural placode were folded and sutured with 6–0 monofilament nylon to reconstruct the thecal sac. Special attention was given to identifying the filum terminal, which, once identified, was freed from any associated attachments. The dura was then dissected from the lumbosacral tissue and fascia and subsequently closed in a watertight fashion (the watertight seal was checked using the Valsalva manoeuvre) (Figure 1).

The initial step consists of dividing the placode from the modified skin. The forceps hold the edge of the placode (b). The placode is detached, and the edges are rolled towards the midline and approximated with several sutures (c). The very thin dura is detached from the edge of the dermis and separated from the adjacent lumbar fascia, and then reflected towards the midline and closed with a continuous suture (d–g). After dural closure, dissection and closure of the skin and the underlying muscle and fascia are performed (h–i).

Most MMCs can be closed using DS. The paravertebral muscles and fascia are mobilised to reinforce the dural suture line, restoring the correct dorsal position relative to the vertebral elements. Bilateral everted paraspinal muscle flaps or fascial flaps can be created. The lumbosacral fascia can be passed over the midline and sutured to the base of the opposite side so that the scar line is not overlaid on the midline, forming overlapping fascial and dermal–adipose planes, which would weaken the resistance and increase the risk of CSF fistula. The skin edges should be approximated to where they meet without tension. Small passive drains can be placed in the superficial plane for 24–48 h to prevent seroma formation, which can be mistaken for CSF leaks (Figure 1). The dissection should be consistent with skin laxity and preserve the subdermal vascular plexus. Depending on the case, when tension does not allow for primary wound closure (DS), local or regional flaps (LF or VY-F) are used.

#### 2.2.1. Limberg Flap (Transposition Flap)

**Figure 2:**
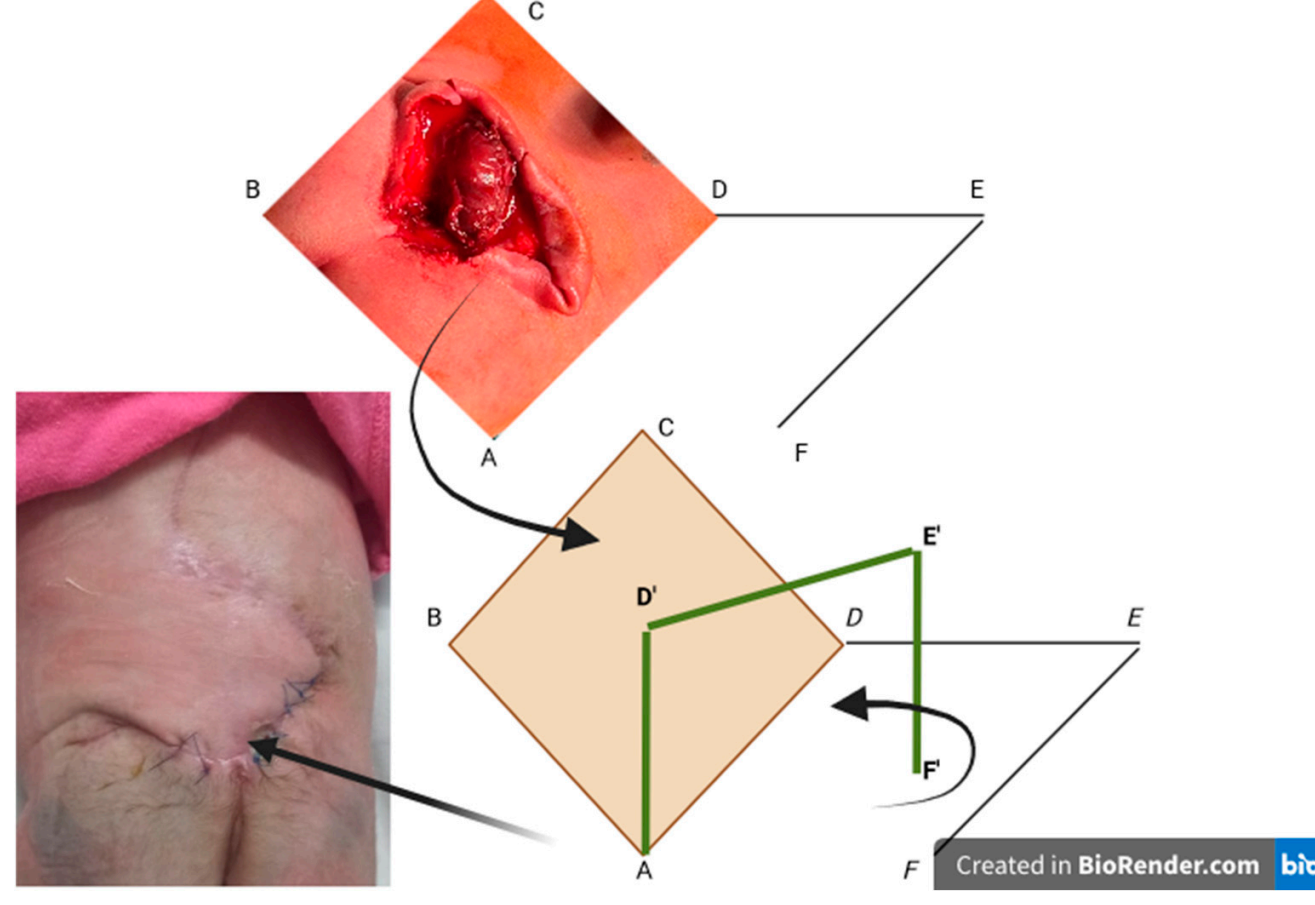
The Limberg flap. The capital letters represent the sides of the rhomboid flap schematically depicted to aid in its explanation. The first black arrow indicates the transition from the rhomboid excision of the myelomeningocele to the graphic transposition of the remaining defect. The second black arrow represents the scheme of the flap transposition, while the last arrow shows the final appearance of this flap. The green line represents the spatial graphic exposure of the flap that is to be raised and transposed into the area of the created rhomboid defect. The green line formed by D’ E’ F’ is the graphic equivalent of DEF. The yellow diamond-shaped area represents the rhomboid defect created after the excision of the myelomeningocele.

The rhomboid flap is a transposition flap that takes the shape of a parallelogram with two 60° angles and two 120° angles, resulting in a defect where all sides and the short diagonal are of the same length (angles may vary depending on the shape of the defect). The larger the angle of the rhomboid, the fewer instances of “dog ear” occur. The flap is designed by extending the short diagonal to a distance equal to one of the sides and drawing an additional line equal in length and parallel to the side adjacent to the defect [13]. To optimise healing, the proximal arm of the flap is marked parallel to the skin tension lines at rest. For flap viability, it is necessary to preserve the dermis and part of the subcutaneous fat to ensure perfusion through the subdermal plexus. Proper dissection allows for wound closure with minimal tension, and extending beyond the base of the flap by dissection avoids forming a raised nodule when transposed (Figure 2).

DE is a direct extension of BD (the short diagonal) with a length equal to BC, to which it will be sutured after rotation. EF is parallel to DA and is equal in size; after rotation, it will be sutured to CD.

The flap used in our series of cases is the fasciocutaneous LF. The technique was completed by inserting a closed active vacuum subcutaneous drainage, which helps prevent hematomas/seromas and provides good adherence of the flap to the underlying tissues. Subcutaneous tissue closure was performed using 3-0/4-0 Vicryl and the skin with separate 4-0/5-0 Prolene sutures (removed after 15–18 days). Superficial drains with negative pressure were placed and removed when the drained amount was less than 8 mL, usually 72 h postoperatively.

#### 2.2.2. VY Advancement Flap

**Figure 3:**
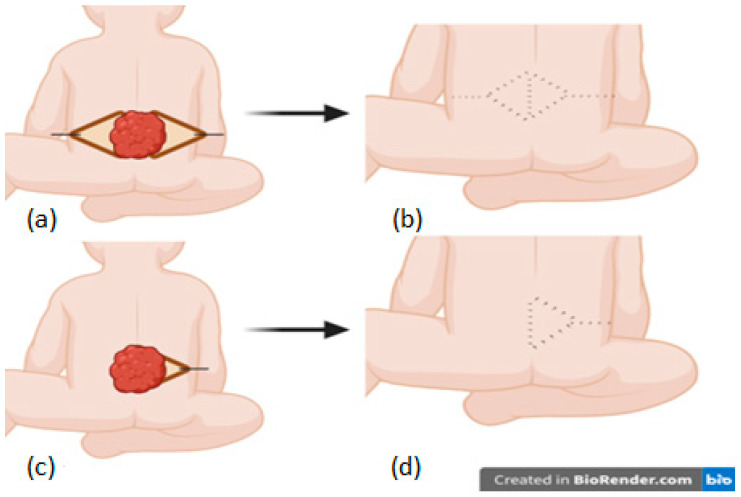
The VY flap: bilateral (**a**,**b**) and unilateral (**c**,**d**).

Unlike the LF, a transposition flap rotating around a pivot point on a horizontal plane, the VY flap is an advancement flap based on two planes of motion (horizontal and vertical) with a pivot point on the vertical plane. The geometric analysis of the VY-F shows that the pivoting plane represents the projection of the triangular flap base in the underlying tissue. The flap design was oriented based on the dimensions of the sides projecting the defect so that advancement was made on the shortest diameter of the lesion. The base size corresponds to the largest diameter of the lesion. Once the height (H) of the triangle was established, the “alpha” angle [14] was estimated between 20° and 40° so that the length of the V was adapted to each case depending on skin elasticity to allow for tension-free suturing of the Y (the angle variation was based on skin elasticity: using a slight angle to obtain a more extended flap to facilitate donor site closure, respectively, a larger angle for a shorter flap when skin elasticity was good) (Figure 3). Once the geometric projection of the flap was created, skin incisions were made vertically to the muscular fascia (deepening the pivoting plane allows for additional advancement to the distal edge of the lesion, becoming a fasciocutaneous/musculocutaneous flap, but these types of flaps were not used in our series to avoid compromising viability, opting instead for a double VY-F in some cases). Additional attention was given to dissection to prevent damaging the paraspinal perforating vessels commonly found around the base of the apical flaps. The procedure was completed by advancing the “V” flaps and closing in a “Y” shape with meticulous hemostasis. Skin closure was performed in a single layer with 3/0 and 4/0 Prolene. Drains were not used for this procedure.

### 2.3. Statistical Analysis

Data processing was performed using SPSS Version 20 (IBM Corp., Armonk, NY, USA), and graphical representation was carried out using Microsoft Word 2023Microsoft Corp., Redmond, WA, USA). Descriptive statistics indicators were calculated as mean, median, mode, skewness and kurtosis parameters, standard deviation, minimum, maximum, and range for numerical data, and for the analysis of categorical or ordinal data, we used the chi-square test. Alpha = 0.05 was the significance threshold, with *p* ≤ 0.05 being statistically significant.

## 3. Results

### 3.1. Patient Population and Clinical Data

**Figure 4:**
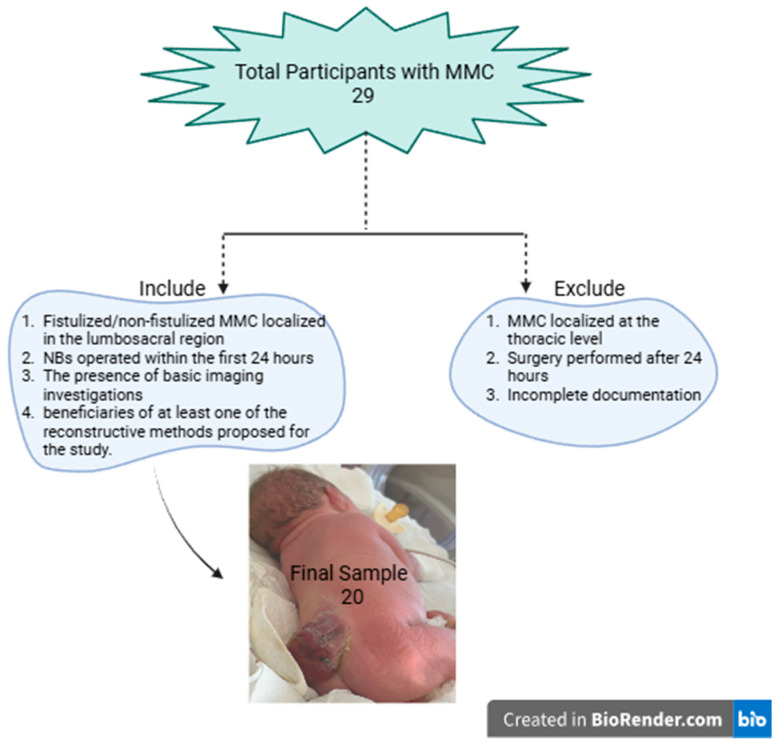
Flow diagram for excluded patients.

Among the NBs with MMC treated in our service between March 2015 and February 2023, 20 cases met the inclusion criteria for this study (Figure 4). A total of 80% (*n* = 16) of the NBs were born at term. Most cases were male (65%, *n* = 13) and had an average hospitalisation duration of 68.95 days (hospitalisation range: 7–360 days). Of the cases studied, 30% (*n* = 6) consisted of fistulated (active CSF leak) MMC (Table 1).

Two patients with thoracic myelomeningocele were not included in this study because both had multiple associated cardiac comorbidities, and one of them died within the first 24 h. Additionally, patients who could not be followed up during the first two years post-surgery due to incomplete documentation were not considered.

Preoperative paraplegia was present in 25% (*n* = 5) of cases, agenesis/hypoplasia of the corpus callosum in 35% (*n* = 7), Chiari 2 malformation in 50% (*n* = 10), and ventriculomegaly in 35% (*n* = 7) of cases.

### 3.2. Type of Closure

**Figure 5:**
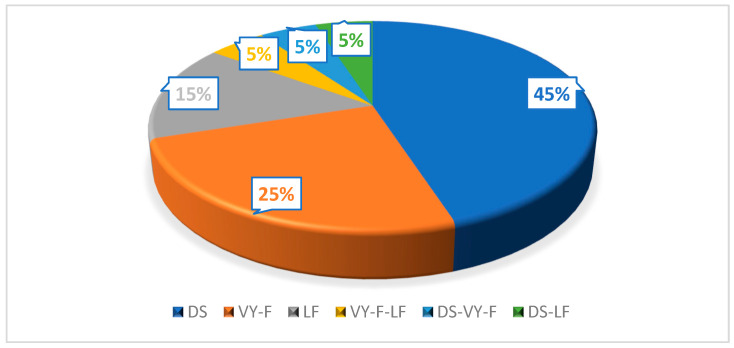
Type of closure used. DS, direct suture; VY-F, VY advancement flap; LF, Limberg flap.

In 45% (*n* = 9) of cases, the remaining defect was covered by DS, in 15% (*n* = 3) by LF, and in 25% (*n* = 5) by VY-F. In 15% (*n* = 3) of cases, combined flaps were decided upon (Figure 5). The average defect size was 3.4 × 3.7 cm.

### 3.3. Post-Procedural Complications

**Figure 6:**
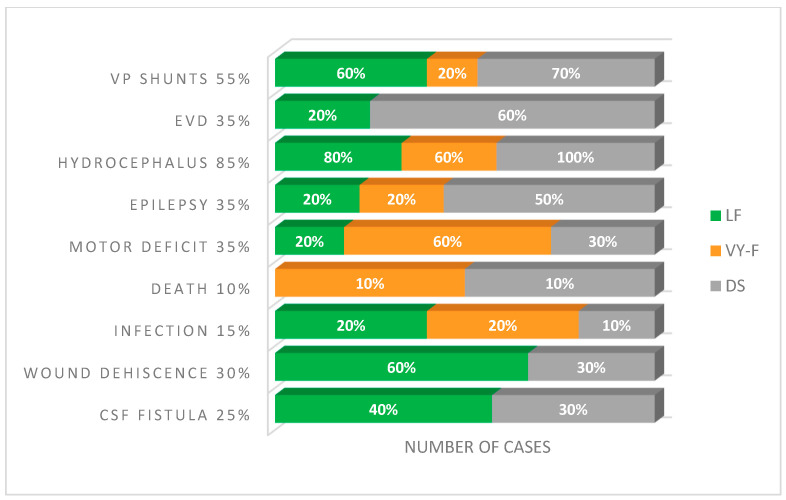
Frequency of complications with the wound closure technology. VP, ventriculoperitoneal; EVD, external ventricular drainage; CSF, cerebrospinal fluid; DS, direct suture; VY-F, VY advancement flap; LF, Limberg flap.

Following the surgical intervention, 10% (two out of twenty) of NBs developed new motor deficits. Among the five (25%) NBs who already had motor deficits before surgery, their conditions either stayed the same or worsened slightly. CSF fistulas were present in 25% (*n* = 5) of NBs, and wound dehiscence was present in 30% (*n* = 6) of cases. Hydrocephalus/ventriculomegaly was present in 85% (*n* = 17) of cases, of which 50% (*n* = 10) developed postoperative hydrocephalus. VP shunt was required in 55% (*n* = 11) of patients. It is necessary to mention that the ventriculoperitoneal shunt rate of the patients included in this study (55%) was excellent compared to that obtained in prenatal surgery, reported at 49%. The slightly insignificant difference between the two values highlights that postnatal surgery is still the standard in MMc treatment [5]. External ventricular drainage (EVD) was required in 35% (*n* = 7) (Figure 6).

### 3.4. Statistical Correlation between Type of Closure and Associated Comorbidities

Regarding the closure technique, 80% (*n* = 4) of those with LF, 20% (*n* = 2) of those with DS, and 0.0% (*n* = 0) of those with VY-F had associated Chiari 2 malformation. Thus, there was a statistically significant association between Chiari 2 malformation and LF closure (*p* = 0.027). There was a statistically significant correlation between the need for EVD and the closure type. Overall, 60% (*n* = 7) in the DS group required EVD; 20% (*n* = 2) in the LF group required EVD, and no patient (0.0%) in the VY-F group required EVD (*p* = 0.041; Table 2). Postoperative intracranial hypertension (IH) was present in 90% (*n* = 10) of the cases in which DS closure was performed, 60% (*n* = 3) in those in which closure was performed, and 20% (*n* = 1) in those in which VY-F closure was performed (*p* = 0.027; Table 2). There was no statistically significant correlation between closure type and the presence of wound dehiscence, infection, CSF fistula, death rate, or the need for a VP shunt (Table 2).

## 4. Discussion

The goal of reconstructive surgery in MMC management is to create a tailored flap for each case, thereby protecting the underlying soft tissues and reducing the incidence of complications. On the other hand, the goal of postnatal surgery is not only to close the defect but also to avoid the inclusion of cysts, dermoid formation, and secondary tethered cord. This can be reached by meticulous postnatal surgery, although not by prenatal surgery, which are precise results of MOMS 1 and MOMS 2 [5,15]. The results obtained through statistical analysis highlight that primary tension-free repair at the suture line can lead to therapeutic success in minor myelomeningocele defects; however, more significant defects present challenges. The VY advancement flap, which utilises the laxity of the nearby skin and preserves the back muscles, is the most effective in minimising both local and general complications.

In our study, we analysed 20 NBs operated on for MMC within the first 24 h after birth. All presented with lumbosacral defects (other cases were excluded). The average hospital stay was two months (ranging from one week to one year), and the average defect size was 3.4 × 3.7 cm (range between 1.5 cm and 6.5 cm). DS closure was performed in 45% of cases, VY-F closure in 25%, LF closure in 15%, and combined flap closure in 15% of patients. The decision on the closure type was made individually, considering the structure and laxity of the remaining defect rather than its size.

A significant statistical correlation was observed between postoperative IH, the need for EVD, and the type of direct closure (60% of patients with DS required EVD, *p* = 0.041, and 90% had IH, *p* = 0.027). Through a detailed analysis of the comorbidities associated with each patient in the group where direct closure was performed (either in combination with another reconstructive method due to its compromise), along with IH and EVD, the presence of Arnold Chiari II malformation was observed in 55% of cases and EVD in 18%, resulting in a total percentage of 73%. Given this data, although there is a statistically significant correlation between the type of closure and IH and the need for EVD placement compared to other techniques, EVD placement must be considered bimodally. The type of suture and the associated predisposing comorbidities (considering their presence in a high percentage of over 70%) correlate with IH and the necessity for EVD placement. Having a larger cohort would have helped clarify this critical detail. The Arnold Chiari malformation is a predisposing associated comorbidity precisely due to its pathogenesis, as hypoplasia of the posterior cranial fossa and occlusion of the jugular foramina lead to cerebellar oedema and, of course, reduced CSF resorption, which ultimately results in hydrocephalus with IH and the need for EVD. The association between Arnold Chiari malformation and IH is supported by different mechanisms that are not yet fully understood [16]

There was no significant statistical correlation between the closure type and the presence of wound dehiscence, infection, or CSF fistula. However, CSF fistula was present in 40% of LF cases and 30% of DS cases, while wound dehiscence was observed in 60% of LF cases and 30% of DS cases. No cases in the VY-F group had CSF fistula or wound dehiscence, indicating that this advancement flap might be the safest method for closing an MMC defect. Wound infection was present in 20% of LF and VY-F cases and 10% of DS cases. All associated complications were successfully resolved with simple auxiliary procedures. Death occurred in 10% of cases.

Regarding local complications such as CSF fistula, wound dehiscence, and infection, the VY advancement flap provides an ideal and safe method for closing an MMC in our study. The VY advancement flap can be the basis for closing significant MMC defects due to its low rate of associated complications. The VY rotation advancement flap, also known as the “butterfly flap”, is described in a case report as a versatile flap that offers the advantage of early healing with a short operative time. This flap was used to cover a 12 × 16 cm defect, demonstrating 99% viability with minimal complications, which were treated conservatively [17].

The randomised controlled Management of Myelomeningocele Study (MOMS) compared the efficacy of prenatal (before 26 weeks of gestation) and postnatal surgeries and was halted early due to the superior outcomes of prenatal surgery. This study demonstrated that in-utero surgery was associated with a 40% reduction in the need for shunt placement and improved mental development and motor function at 30 months. However, it also increased the risks of premature birth, intraoperative complications, and uterine dehiscence at birth [15,18].

Despite these findings, fetal surgery has not yet been implemented in many institutions, underscoring the relevance of postnatal management of NBs with MMC, including the surgical closure techniques used and associated secondary complications. Furthermore, prenatal surgery should not be implemented further without more studies because we currently do not have sufficient evidence to recommend in utero repair for children born with spina bifida [2]

Various uses of musculocutaneous flaps of the latissimus dorsi for closing MMC have been reported, including bilateral musculocutaneous flaps, extended bilateral flaps with gluteal fasciocutaneous flaps, LFs, distal-based and reversible flaps, or a combination of latissimus dorsi and gluteus maximus musculocutaneous flaps for lower sacral defects [19,20].

Patel et al. performed soft tissue defect reconstruction using paraspinous fascial flaps. After neurosurgical repair, they elevated the paraspinous fascia from lateral to medial. The raised fascial flaps were turned over and sutured at the midline [21]. The paraspinous fascia provides a strong, vascularised, and tension-free coverage over the dura mater, reinforcing the midline and reducing the risk of CSF fistula. It also eliminates the need for significant muscle flaps, which are more complicated to dissect. This study supports performing a linear, midline DS to facilitate exposure for potential secondary spinal procedures, such as the need for posterior fusion for scoliosis. In our series of cases, DS was associated with the highest rate of complications, although it was the type of suture used in 45% of cases.

Shim et al. presented 14 cases of MMC operated on in their department over 10 years; 86% of cases underwent DS of the wound, while LF was performed in 14% of patients. Hydrocephalus was present in both patients who underwent LF and in only 41% of those who underwent DS. LF allows for the use of redundant skin from the scapular area to the upper thoracic region, which helps cover the defect without generating excessive tension, preserves the back muscles, and allows patients to maintain correct trunk posture as they grow. All sides of a rhomboid defect can be used as flaps in LF, meaning four flaps can be raised simultaneously to cover the defect, and circulation is stable in the centre of the flap, aiding in the proper healing of the defect. On the other hand, wound dehiscence was present in 100% of cases in which LF was performed [11]. Similarly, in our series of cases, wound dehiscence was most frequently present in LF cases, at a rate of 80% and fistula, at a rate of 40%. Even with the aforementioned advantages, the decision to perform LF closure should, in our opinion, be a reserved one, not a first choice. We note that in our study, a fasciocutaneous LF closure was performed. The consideration for opting for a fasciocutaneous flap instead of a dermo adipose one is to obtain a better-vascularised flap by supplementing the randomised vascularisation of the fascial plexus. The fasciocutaneous flap is more suitable for deep and complex defects that require adequate structural support and good vascularisation.

Lobo and Nayak conducted a prospective study involving 22 patients, using a VY-F fasciocutaneous flap for 59% and DS for the remaining 41%. Postoperatively, all nine (100%) patients who underwent direct closure presented with CSF fistula, and six (66.6%) of them had wound dehiscence, while one (11.1%) of the NBs died. Only three (23.07%) had CSF fistula among the patients with VY-F, and none (0.0%) had wound dehiscence. Regarding the average closure time, DS was completed in 120 min, while VY-F took 190.7 min, thus subjecting NBs to prolonged anaesthesia, and blood loss was higher in the VYF group since cauterisation was used less to preserve blood supply [10]. Similarly, in our study, no patient who underwent VY-F had CSF fistula or wound dehiscence, reducing the hospitalisation time of the NBs, demonstrating the superiority of this closure type compared to DS. On the other hand, our results and this study highlight the importance of performing an individualised flap based on skin laxity and the anatomical conformation of the NBs rather than the defect size. It should also be noted that in a fasciocutaneous flap, the muscle structure remains intact, thus maintaining its function of posture and stability.

Ulusoy et al. conducted a study involving 10 NBs using a modified bilateral VY advancement flap closure technique. After marking bilateral VY advancement flaps on the children’s backs, the height of the V flaps was set at 1.5 to 2 times the diameter of the defect, with the branches of the V slightly curved outward to create wider flaps and extensions than the standard. The wide extensions of the V flaps surrounded the defect from above and below. Skin incisions were made vertically to the muscle fascia, with limited and additional undermining to elevate the upper and lower extensions of the V flaps over a distance equal to the radius of the defect. Meticulous dissection was essential to preserve vascularisation. Instead of sliding the “V” flaps into the defect for “Y” type advancement and vertical closure on the midline, the apical extensions of the “V” flaps were mobilised and evaluated for the best possible closure, considering the size, shape, and location of the defect. The apical extensions were transposed and supported by standard VY advancement of the bilateral flaps to achieve tension-free closure. After adapting the apical flaps to the defect site, the operation was completed by advancing the “V” flaps and closing in the “Y” shape [13].

Thus, since the apical extensions of the “V” flaps were well-vascularised, the optimal intraoperative decision was made to customise the flap based on size, shape, and location. The flap was individualised to the region’s anatomy—the apical extensions of the “V” flaps were elevated based on paraspinous perforators. The study results show that all defects were successfully closed, and there were no signs of ischemia or dehiscence. The only complication among the 10 patients was a CSF fistula (10%), which was resolved by revision. The results of this study support our findings that VY-F closure is superior to other types. We observed no cases of wound dehiscence, CSF fistula, or vascular distress, and VY-F ranked first in terms of postoperative incidence of IH. The decision to choose a classic VY advancement flap over a modified one was mainly based on the peculiarities of the cases studied. Covering a very large defect requires flap customisation for optimal closure. In our study, it was not considered necessary to use variants of the VY-F, as no difficulties or aesthetic issues were encountered in closing the defects while maximising flap viability by avoiding additional incisions in their apical portions.

The major limitation of this study is the relatively small number of patients since this study was conducted in a single centre. A multicentre research would have allowed for better coverage of MMC pathology, with a broader selection of patients and a much more uniform distribution across different subpopulations for subsequent statistical evaluation. Another limitation is the absence of sophisticated multivariate analysis, which is essential to confirming the presence of these associations. Due to the available medical techniques—genetic, imaging, and infectious diagnostics—the correlation between epilepsy and cortical malformations could not be established. Considering the data available in the literature and the aim of this study, which was to identify the reconstructive method with minimal complications, the role of epilepsy as a postoperative complication of MMC was investigated without resulting in a statistically significant relationship between these variables.

Additionally, there could be unknown selection bias due to the non-randomised case selection. Additionally, due to the retrospective nature of this study, precise information related to the exact aspects of the lesion, such as photographs or the time required for performing DS/LF/VY-F, is missing, which could have influenced the current results and conclusions.

## 5. Conclusions

Our study indicates that VY-F is the safest method for closing MMC defects, minimising the risk of postoperative complications. DS was associated with the highest rate of severe complications. At the same time, LF, though more complex, provided adequate coverage for significant defects but was also associated with a higher rate of complications. Another critical aspect of this study is that the choice of closure technique should be based on the laxity of the defect and the specific anatomical conditions of the patient, not necessarily the defect size.

Our study contributes to the understanding of the surgical management of MMC and emphasises the importance of selecting an appropriate closure technique to maximise clinical outcomes and minimise complications.

## Figures and Tables

**Table 1:** Patient data.

No.	Size of the Defect (Height/Width) cm	MMC Fistulated	Cranio-Cerebral Malformations Associated	Duration of Hospitalisation (No of Days)	Type of Closure
1	3.5 × 6.5	–	Ventriculomegaly	30	DS
2	1.5 × 2	–	–	30	DS
3	2 × 3.5	+	Ventriculomegaly, Paraparesis	12	VY-F
4	4 × 5	–	Chiari 2, Paraparesis	45	DS
5	1.5 × 3	+	Chiari 2, Agenesis of the corpus callosum, Ventriculomegaly	60	DS
6	6 × 5	–	–	35	DS
7	3 × 3	–	Chiari 2, Agenesis of the corpus callosum, Paraparesis	37	VY-F
8	4 × 4.5	–		7	DS
9	2 × 2	+	Chiari 2, Ventriculomegaly	30	L-F
10	3 × 4	–	–	260	VY-F
11	4 × 4.5	–	Chiari 2, Agenesis of the corpus callosum	60	DS
12	6 × 5	+	Chiari 2, Agenesis of the corpus callosum, Ventriculomegaly	25	L-F
13	3 × 2	–	Hypoplasia of the corpus callosum	20	VY-F
14	5 × 3.5	–	Ventriculomegaly	60	DS
15	3 × 2	–	–	46	L-F
16	5 × 3.5	–	Ventriculomegaly	360	VY-F
17	3.5 × 4	–	Chiari 2,	90	DS
18	2 × 2	+	Chiari 2, Hypoplasia of the corpus callosum, Paraparesis	38	DSVYF
19	4 × 4.5	–	Chiari 2, Paraparesis	60	VY-F- LF
20	2 × 4.5	+	Chiari 2, Hypoplasia of the corpus callosum	74	DS- LF

VY-F was performed after DS failure; MMC, myelomeningocele; DS, direct suture; VY-F, VY advancement flap; LF, Limberg flap; +, present; –, absent.

**Table 2:** Complications and surgical measures post-MMC intervention in relation to the type of closure performed.

Motor Deficit									
	LF	VY-F	DS	Pearson Chi-Square Test			Likelihood-Ratio Chi-Square Test		
				Chi-Square	DF	*p*-Value	Chi-Square	DF	*p*-Value
No	80.0%	40.0%	70.0%	1.978 a	2	0.372	1.946	2	0.378
Yes	20.0%	60.0%	30.0%	a. Five cells (83.3%) have expected count of less than 5. The minimum expected count is 1.75					
Epilepsy									
	LF	VY-F	DS	Pearson chi-square test			Likelihood-ratio chi-square test		
				Chi-Square	DF	*p*-Value	Chi-Square	DF	*p*-Value
No	80.0%	80.0%	50.0%	1.978 a	2	0.372	2.027	2	0.363
Yes	20.0%	20.0%	50.0%	a. Five cells (83.3%) have expected count of less than 5. The minimum expected count is 1.75					
Hydrocephalus									
	LF	VY-F	DS	Pearson chi-square test			Likelihood-ratio chi-square test		
				Chi-Square	DF	*p*-Value	Chi-Square	DF	*p*-Value
No	20.0%	40.0%	0.0%	4.314 a	2	0.116	5.174	2	0.075
Yes	80.0%	60.0%	100.0%	a. Five cells (83.3%) have an expected count of less than 5. The minimum expected count is 0.75					
EVD									
	LF	VY-F	DS	Pearson chi-square test			Likelihood-ratio chi-square test		
				Chi-Square	DF	*p*-Value	Chi-Square	DF	*p*-Value
No	80.0%	100.0%	40.0%	5.934 a	2	0.041	7.434	2	0.024
Yes	20.0%	0.0%	60.0%	a. Five cells (83.3%) have expected count of less than 5. The minimum expected count is 1.75					
VP shunt									
	LF	VY-F	DS	Pearson chi-square test			Likelihood-ratio chi-square test		
				Chi-Square	DF	*p*-Value	Chi-Square	DF	*p*-Value
No	40.0%	80.0%	30.0%	3.434 a	2	0.180	3.574	2	0.167
Yes	60.0%	20.0%	70.0%	a. Five cells (83.3%) have expected count of less than 5. The minimum expected count is 1.75					
ICH									
	LF	VY-F	DS	Pearson chi-square test			Likelihood-ratio chi-square test		
				Chi-Square	DF	*p*-Value	Chi-Square	DF	*p*-Value
No	40.0%	80.0%	10.0%	7.253 a	2	0.027	7.662	2	0.022
Yes	60.0%	20.0%	90.0%	a. Five cells (83.3%) have expected count of less than 5. The minimum expected count is 1.75					
Ventriculomegaly									
	LF	VY-F	DS	Pearson chi-square test			Likelihood-ratio chi-square test		
				Chi-Square	DF	*p*-Value	Chi-Square	DF	*p*-Value
No	60.0%	60.0%	100.0%	5.000 a	2	0.082	6.556	2	0.038
Yes	40.0%	40.0%	0.0%	a. Five cells (83.3%) have expected count of less than 5. The minimum expected count is 1.00					
CSF Fistula									
	LF	VY-F	DS	Pearson chi-square test			Likelihood-ratio chi-square test		
				Chi-Square	DF	*p*-Value	Chi-Square	DF	*p*-Value
No	60.0%	100.0%	70.0%	2.400 a	2	0.301	3.546	2	0.170
Yes	40.0%	0.0%	30.0%	a. Five cells (83.3%) have expected count of less than 5. The minimum expected count is 1.25					
Wound Dehiscence									
	LF	VY-F	DS	Pearson chi-square test			Likelihood-ratio chi-square test		
				Chi-Square	DF	*p*-Value	Chi-Square	DF	*p*-Value
No	40.0%	100.0%	70.0%	4.286 a	2	0.117	5.487	2	0.064
Yes	60.0%	0.0%	30.0%	a. Five cells (83.3%) have expected count of less than 5. The minimum expected count is 1.50					
Infection									
	LF	VY-F	DS	Pearson chi-square test			Likelihood-ratio chi-square test		
				Chi-Square	DF	*p*-Value	Chi-Square	DF	*p*-Value
No	80.0%	80.0%	90.0%	0.392 a	2	0.822	0.399	2	0.819
Yes	20.0%	20.0%	10.0%	a. Five cells (83.3%) have expected count of less than 5. The minimum expected count is 0.75					
Death									
	LF	VY-F	DS	Pearson chi-square test			Likelihood-ratio chi-square test		
				Chi-Square	DF	*p*-Value	Chi-Square	DF	*p*-Value
No	100.0%	80.0%	90.0%	1.111a	2	0.574	1.498	2	0.473
Yes	0.0%	10.0%	10.0%	a. Five cells (83.3%) have expected count of less than 5. The minimum expected count is 0.50					

DS, direct suture; VY-F, VY advancement flap; FL, Limberg flap; EVD, external ventricular drainage; VP, ventriculoperitoneal; IH, intracranial hypertension; CSF, cerebrospinal fluid; MMC, myelomeningocele. Death occurred in two NB patients. One benefited from a VY flap, while the other initially had direct closure; however, due to local and general complications, another intervention was necessary, during which the defect was closed using a VY advancement flap.

## Data Availability

The raw data supporting the conclusions of this article will be made available by the authors on request.
